# Match demands of female team sports: a scoping review

**DOI:** 10.5114/biolsport.2024.129476

**Published:** 2023-07-24

**Authors:** María L. Pérez Armendáriz, Konstaninos Spyrou, Pedro E. Alcaraz

**Affiliations:** 1 UCAM Research Center for High Performance Sport, UCAM Universidad Católica de Murcia, Murcia, Spain; 2 Facultad de Deporte, UCAM Universidad Católica de Murcia, Murcia, Spain; 3 Strength and Conditioning Society, Murcia, Spain

**Keywords:** Women, External load, Performance, Tracking system

## Abstract

This scoping review aimed to characterize and quantify the external load demands of professional female team sports, in terms of total distance [TD], moderate-speed [MSR] and high-speed running [HSR], sprint, accelerations [ACC], and decelerations [DEC]. A search was conducted in PubMed, Scopus, and Web of Science until 15/04/2023. The Risk of Bias Assessment Tool for Nonrandomized Studies (RoBANS) was used. Eighty-six articles were eligible for inclusion in this review, with 40 in soccer, 23 in rugby (6 rugby union, 3 rugby league, and 14 rugby sevens), 8 in field hockey, 8 in basketball, 6 in handball, and 1 in futsal. Soccer is the most investigated sport, and players perform ~9500 m TD, of which ~580 m is performed in HSR, and with a great number of ACC, DEC, and sprints. Rugby league and union players cover a greater distance (~5450 m) when compared to rugby sevens (~1550 m); however, rugby sevens is more demanding in terms of high-intensity actions. Field hockey players perform ~5400 m TD with high-intensity and sprint actions. Women’s indoor sports are less studied, and basketball players cover ~5300 m TD, of which 7% is performed in MSR. Handball players perform ~3500 m TD and cover ~423 m in MSR and ~141 m in HSR, and futsal players perform ~5 m × min^−1^ in HSR and they do a great number of high-intensity activities (HSR, ACC, and DEC). Considering the high physical demands experienced by female athletes, professionals could use the present results for training and return to competition schedules.

## INTRODUCTION

Female team sports’ participation and popularity have increased considerably in the last decade [1]. This increase has attracted more sports scientists, strength and conditioning coaches, and medical staff into the field [1–4]. However, a recent scoping review [5] about external load monitoring with wearable technology from 2015 to 2020 reported that only 16.2% of the investigations were carried out with female athletes, compared to 80.6% with male counterparts. Moreover, current sports performance methods and strategies in female team sports are often supported by evidence derived from male athletes [3, 4]. Consequently, sport practitioners should understand better the physiological and mechanical demands during match play in female team sports [6].

The external load represents the basic measurement of a monitoring system [7] and expresses the activities performed by an athlete [8] independently of its internal characteristics (i.e., internal load) [9]. The consensus statement of the International Olympic Committee on load in sports and risk of injury states that a successful training load monitoring system is fundamental to ensure the adaptation to stress, maximize physical performance, and possibly minimize the risk of injury [10]. In team sports, physical activity can be registered by different tracking systems, such as global positioning systems (GPS), local positioning systems (LPS), inertial measurement units (IMU), and time-motion analysis (TMA) [11–16]. Each system has its limitations; therefore a pragmatic and systematic approach to data collection, analysis, and interpretation is necessary [11]. Total distance (TD) is generally used as an indicator of overall training volume [11, 17], while high-speed running (HSR), acceleration (ACC) and deceleration (DEC) actions refer to a neuromuscular type of loading, which is likely more related to injury risk [18–20], and lastly Player Load (Pload) provides an estimate of the total cost of movement actions [17, 21].

The analysis of the physical demands during matches is an essential element for broadening knowledge of the stress that players experience at this level [22]. This information may help professionals to design appropriate training and return to play programme sessions regarding the match [16, 22]. For example, Taylor et al. [16] analysed the demands of athletes in both men and women in different team sports (soccer, basketball, handball, futsal, and field hockey) and categories (elite, sub-elite and junior), where only 10 studies were found in elite female players (soccer = 5, basketball = 2, handball = 2, field hockey = 1). Therefore, more research, characterizing the match demands in female team sports, to implement further evidence-based practices, is warranted.

To the authors’ knowledge, this is the first study to review the professional female athletes’ match demands, collected by external load from six different team sports (soccer, rugby, field hockey, basketball, handball, and futsal). The aim of this scoping review was to characterize and quantify the demands of external load (i.e., TD, moderatespeed running [MSR], HSR, sprint, ACC, DEC, and Pload) in professional female multi-directional team sports and highlight the importance of research on female sport [4, 23].

## MATERIALS AND METHODS

### Protocol and registration

The scoping review protocol was preliminarily submitted and published on the Open Science Framework, with the registration number 10.17605/OSF.IO/E4H9M on 29^th^ April 2023.

### Study design

The present study is a scoping review focused on the match demands of professional women’s team sports (i.e., soccer, rugby, field hockey, basketball, handball, and futsal) measured with a tracking system. The review was carried out in accordance with the recommendations for Systematic Reviews and Meta-Analyses (PRISMA) [24] and did not require institutional review board approval.

### Data sources and searches

A scoping review of the literature was performed using three different online databases – PubMed, Scopus, and Web of Science – until April 15^th^, 2023. In order to ensure that all research related to this topic was identified, a broad and general search was carried out, searching for the following terms: [(“match analysis” OR “GPS” OR “demands” OR “external load”) AND (“basketball”/ “field hockey”/ “football OR soccer”/ “handball”/ “rugby”/ “futsal”) AND (“female” OR “women”) NOT “male”], to ensure that all studies related to this topic were identified, and the search was repeated for each sport individually. This search was performed by two authors (MLPA and KS), and search results were uploaded to reference management software (Zotero) where duplicates were automatically removed. All titles and abstracts of all remaining studies were screened by two authors (MLPA and KS) using the eligibility criteria below. Any disagreements about study inclusion/exclusion that could not be resolved between the two authors were decided by a third party (PEA).

### Eligibility criteria

Studies were eligible for inclusion if they met the following criteria: 1) a sample of highly trained and competitive/professional female athletes according to classification of levels of competition adapted from Russell et al. [25], aged > 18 years; 2) competing in soccer, rugby, field hockey, basketball, handball, and futsal; and lastly 3) incorporating tracking systems (i.e., GPS, LPS, TMA or IMU) and analysing some external load variables (i.e., TD, distance per zone, ACC, DEC, Pload).

Studies were excluded if they: 1) did not include original data; 2) were not available in English and full text; 3) reported simulated games and/or drills; and 4) scored < 8 in methodological quality assessment.

### Study selection

The initial search was carried out by two researchers (MLPA and KS). After the elimination of duplicates, an intensive review of all titles and abstracts obtained was completed and those not related to the review’s topic were discarded. The full version of the remaining articles was read. All studies not meeting the inclusion criteria were excluded.

### Data extraction

Data were extracted into a custom-made Microsoft Excel sheet (2007) by one author (MLPA), with two other authors (KS and PEA) checking for the accuracy. The results were selected with the following order: participant’s information (i.e., sample size, age, height, weight), number of matches, country, equipment used (i.e., device brand, model details, sampling frequency (Hz), according to recommendations for the collecting, processing and reporting of data from GPS devices [26] external load metrics (i.e., TD, distance at MSR [12.6–19.8 km · h^−1^], HSR [19.8–25.2 km · h^−1^], and sprinting [≥ 25.2 km · h^−1^], ACC, DEC, Pload). The mean and standard deviation (SD) were extracted for all the variables, and presented as full match-play. Intensity thresholds for ACC and DEC were presented. A meta-analysis was not performed due to the heterogeneous nature of sport specific study designs and inability to pool data.

### Risk of bias

The risk of bias was evaluated independently by two authors (MLPA and KS), who reanalysed the process in cases of disagreement. If a consensus was not reached, a final decision was made by a third author (PEA). The Risk of Bias Assessment Tool for Nonrandomized Studies (RoBANS) was utilized to evaluate the included studies’ risk of bias, as it has demonstrated moderate reliability and good feasibility and validity [27]. The tool comprises six domains, which are the selection of participants, confounding variables, measurement of exposure, blinding of outcome assessments, incomplete outcome data, and selective outcome reporting, and these domains are classified as ‘low’, ‘high’, or ‘unclear’ risk of bias [27].

### Methodological quality assessment

The methodological quality of the included studies was assessed by two researchers (MLPA and KS) using the modified Downs & Black [28] evaluation scale. Of the total 27 criteria, 12 were used according to the study’s design (i.e., descriptive), as observed in similar systematic reviews [13, 14, 29].

## RESULTS

### Search results

**FIG. 1:**
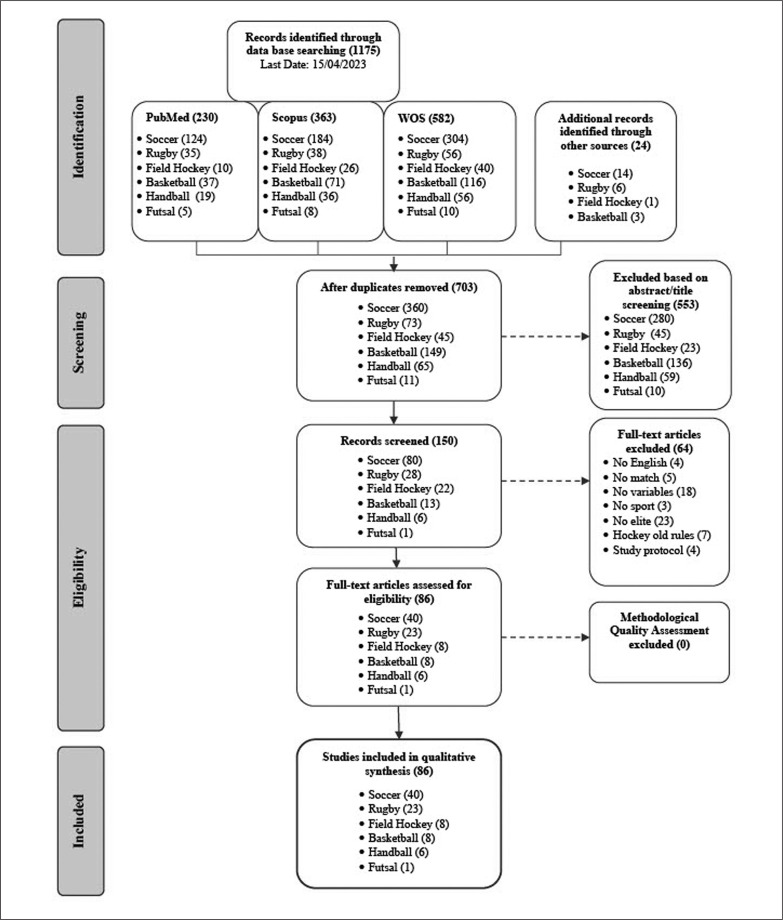
Flow diagram.

Figure 1 depicts the PRISMA flow diagram of the search and selection process. The initial databases yielded 1175 studies, and 24 additional records were added through other sources. After duplicate removal, 703 articles remained. Upon title and abstract screening, 150 were left for full-text review. Of the 150 articles reviewed, 86 met the inclusion criteria in this systematic review: 40 on soccer [30–70], 23 on rugby [71–93], 8 on field hockey [94–101], 8 on basketball [102–109], 6 on handball [110–115], and 1 on futsal [116].

### Risk of bias

The results of the risk of bias assessment can be seen in Table 1. Overall the confounding variables were unclear in the majority (64%) of the articles. This is because contextual factors (e.g., sleep, nutrition, training, climate) were not reported or not controlled for. The risk of bias in the measurement of exposure was unclear in 16% of the articles and high in 13%, as assessments of demands were not conducted over a considerable period of time (> 4 matches) or in relation to the reliability of the measurement instrument. All included studies had a low risk of bias in the selection of participants.

**TABLE 1:** Risk of bias assessment of non-randomized studies.

Author (year)	Selection of participants	Confounding variables	Measurement of exposure	Blinding of outcome assessments	Incomplete outcome data	Selective outcome reporting
Andersson et al. (2010) [51]	*Low*	*Unclear*	*Unclear*	*Low*	*Low*	*Low*

Bradley et al. (2014) [60]	*Low*	*Unclear*	*Low*	*Low*	*Low*	*Low*

Busbridge et al. (2020) [92]	*Low*	*Unclear*	*Low*	*Low*	*Low*	*Low*

Callanan et al. (2021) [73]	*Low*	*Low*	*Low*	*Low*	*Low*	*Low*

Choi et al. (2020) [96]	*Low*	*Unclear*	*Low*	*Low*	*Low*	*Low*

Choi et al. (2022) [67]	*Low*	*Unclear*	*Low*	*Low*	*Low*	*Low*

Clarke et al. (2014a) [88]	*Low*	*Low*	*High*	*Low*	*Low*	*Low*

Clarke et al. (2014b) [86]	*Low*	*Unclear*	*Low*	*Low*	*Low*	*Low*

Clarke et al. (2015) [85]	*Low*	*Unclear*	*Low*	*Low*	*Unclear*	*Low*

Clarke et al. (2017) [90]	*Low*	*Unclear*	*Low*	*Low*	*Low*	*Low*

Conte et al. (2015) [105]	*Low*	*Unclear*	*High*	*Low*	*Low*	*Low*

Conte et al. (2022) [77]	*Low*	*Unclear*	*Low*	*Low*	*Low*	*Low*

Datson et al. (2017) [47]	*Low*	*Unclear*	*Unclear*	*Low*	*Low*	*Low*

Datson et al. (2019) [61]	*Low*	*Unclear*	*High*	*Low*	*Low*	*Low*

Del coso et al. (2013) [89]	*Low*	*Low*	*Unclear*	*Low*	*Low*	*Low*

Delextrat et al. (2017) [104]	*Low*	*Unclear*	*Unclear*	*Low*	*Low*	*Low*

Delextrat et al. (2012) [108]	*Low*	*Low*	*High*	*Low*	*Low*	*Low*

Delves et al. (2021) [101]	*Low*	*Unclear*	*Low*	*Low*	*Low*	*Low*

DeWitt et al. (2018) [45]	*Low*	*Low*	*Low*	*Low*	*Low*	*Low*

Diaz-Seradilla et al. (2022) [58]	*Low*	*Unclear*	*Low*	*Low*	*Low*	*Low*

Doeven et al. (2019) [91]	*Low*	*Low*	*Low*	*Low*	*Low*	*Low*

Emmonds et al. (2020) [75]	*Low*	*Unclear*	*Unclear*	*Low*	*Low*	*Low*

Fernandes et al. (2022) [56]	*Low*	*Low*	*Low*	*Low*	*Low*	*Low*

Gabbett et al. (2008) [62]	*Low*	*Unclear*	*Unclear*	*Low*	*Low*	*Low*

García-Ceberino et al. (2022) [57]	*Low*	*Low*	*Unclear*	*Low*	*Low*	*Low*

Gonçalves et al. (2021) [54]	*Low*	*Unclear*	*Low*	*Unclear*	*Low*	*Low*

Goodale et al. (2006) [84]	*Low*	*Unclear*	*Unclear*	*Low*	*Low*	*Low*

Griffin et al. (2021) [38]	*Low*	*Unclear*	*Low*	*Low*	*Low*	*Low*

Hewitt et al. (2014) [50]	*Low*	*Unclear*	*Low*	*Low*	*Low*	*Low*

Julian et al. (2021) [37]	*Low*	*Low*	*Unclear*	*Low*	*Low*	*Low*

Kapteijns et al. (2021) [94]	*Low*	*Unclear*	*Low*	*Low*	*Low*	*Low*

Kim et al. (2016) [99]	*Low*	*Unclear*	*High*	*Low*	*Low*	*Low*

Kniubaite et al. (2019) [110]	*Low*	*Low*	*Low*	*Low*	*Low*	*Low*

Kobal et al. (2022a) [69]	*Low*	*Unclear*	*Low*	*Low*	*Low*	*Low*

Kobal et al. (2022b) [68]	*Low*	*Unclear*	*Low*	*Low*	*Low*	*Low*

Krustrup et al. (2005) [53]	*Low*	*Unclear*	*Low*	*Low*	*Low*	*Low*

Krustrup et al. (2021) [36]	*Low*	*Low*	*Low*	*Low*	*Low*	*Low*

Luteberget et al. (2016) [111]	*Low*	*Unclear*	*Low*	*Low*	*Low*	*Low*

Luteberget et al. (2017) [112]	*Low*	*Low*	*Low*	*Low*	*Low*	*Low*

Malone et al. (2020) [78]	*Low*	*Unclear*	*Low*	*Low*	*Low*	*Low*

Manchado et al. (2013) [115]	*Low*	*Unclear*	*High*	*Low*	*Low*	*Low*

Mara et al. (2016) [63]	*Low*	*Unclear*	*Low*	*Low*	*Low*	*Low*

Mara et al. (2017) [46]	*Low*	*Unclear*	*Low*	*Low*	*Low*	*Low*

McGuinness et al. (2018) [100]	*Low*	*Low*	*Low*	*Low*	*Low*	*Low*

McMahon et al. (2019) [98]	*Low*	*Unclear*	*Low*	*Low*	*Low*	*Low*

Meylan et al. (2016) [49]	*Low*	*Unclear*	*Low*	*Low*	*Low*	*Low*

Michalsik et al. (2014) [114]	*Low*	*Low*	*Unclear*	*Low*	*Low*	*Low*

Misseldine et al. (2018) [79]	*Low*	*Low*	*Low*	*Low*	*Low*	*Low*

Mohr et al. (2008) [52]	*Low*	*Unclear*	*High*	*Low*	*Low*	*Low*

Morencos et al. (2019) [97]	*Low*	*Unclear*	*Low*	*Low*	*Low*	*Low*

Nakamura et al. (2017) [64]	*Low*	*Unclear*	*Unclear*	*Low*	*Low*	*Low*

Newans et al. (2021) [72]	*Low*	*Unclear*	*Low*	*Low*	*Low*	*Low*

Nolan et al. (2023) [93]	*Low*	*Unclear*	*Low*	*Low*	*Low*	*Low*

Oliva Lozano et al. (2021) [116]	*Low*	*Unclear*	*Low*	*Low*	*Low*	*Low*

Palmer et al. (2021) [102]	*Low*	*Low*	*Low*	*Low*	*Low*	*Low*

Palmer et al. (2022) [107]	*Low*	*Low*	*Low*	*Low*	*Low*	*Low*

Panduro et al. (2021) [34]	*Low*	*Unclear*	*Unclear*	*Low*	*Low*	*Low*

Park et al. (2018) [42]	*Low*	*Low*	*Low*	*Low*	*Low*	*Unclear*

Portillo et al. (2014) [82]	*Low*	*Low*	*Low*	*Low*	*Low*	*Low*

Principe et al. (2021) [35]	*Low*	*Unclear*	*Low*	*Unclear*	*Low*	*Low*

Quinn et al. (2019) [87]	*Low*	*Unclear*	*Low*	*Low*	*Low*	*Low*

Ramos et al. (2017) [65]	*Low*	*Unclear*	*Low*	*Low*	*Low*	*Low*

Ramos et al. (2019a) [41]	*Low*	*Unclear*	*Low*	*Low*	*Low*	*Low*

Ramos et al. (2019b) [66]	*Low*	*Low*	*Low*	*Low*	*Low*	*Low*

Reina et al. (2022) [109]	*Low*	*Low*	*Low*	*High*	*Low*	*Low*

Reyneke et al. (2018) [80]	*Low*	*Unclear*	*Low*	*Low*	*Low*	*Low*

Romero-Moraleda et al. (2021) [33]	*Low*	*Low*	*Low*	*Low*	*Low*	*Low*

Sánchez-Migallón et al. (2020) [95]	*Low*	*Low*	*High*	*Low*	*Low*	*Low*

Scanlan et al. (2012) [106]	*Low*	*Low*	*High*	*Low*	*Low*	*Low*

Scott et al. (2020a) [39]	*Low*	*Unclear*	*Low*	*Low*	*Low*	*Low*

Scott et al. (2020b) [40]	*Low*	*Unclear*	*Unclear*	*Low*	*Low*	*Low*

Sheppy et al. (2020) [74]	*Low*	*Unclear*	*Low*	*Low*	*Low*	*Low*

Stauton et al. (2018) [103]	*Low*	*Unclear*	*Low*	*Low*	*Low*	*Low*

Suarez-Arrones et al. (2014) [76]	*Low*	*Low*	*High*	*Low*	*Low*	*Low*

Suarez-Arrones et al. (2012) [83]	*Low*	*Low*	*High*	*Low*	*Low*	*Low*

Trewin et al. (2017) [43]	*Low*	*Low*	*Unclear*	*Low*	*Low*	*Low*

Trewin et al. (2018) [48]	*Low*	*Low*	*Low*	*Low*	*Low*	*Low*

Vescovi et al. (2012) [44]	*Low*	*Low*	*Low*	*Low*	*Low*	*Low*

Vescovi et al. (2015) [81]	*Low*	*Unclear*	*Low*	*Low*	*Low*	*Low*

Vescovi et al. (2019) [55]	*Low*	*Unclear*	*Low*	*Low*	*Low*	*Low*

Villaseca-Vicuña et al. (2021) [32]	*Low*	*Low*	*Low*	*Low*	*Low*	*Low*

Villaseca-Vicuña et al. (2023) [70]	*Low*	*Low*	*Low*	*Low*	*Low*	*Low*

Wik et al. (2016) [113]	*Low*	*Unclear*	*Low*	*Low*	*Low*	*Low*

Winther et al. (2021) [31]	*Low*	*Unclear*	*Low*	*Low*	*Low*	*Low*

Woodhouse et al. (2021) [71]	*Low*	*Unclear*	*Low*	*Low*	*Low*	*Low*

Yousefian et al. (2021) [30]	*Low*	*Unclear*	*Low*	*Low*	*Low*	*Low*

### Soccer

Table 2 presents the match demands, anthropometric data and origin of players in soccer. The match demands were collected by TMA (n = 9) and GPS devices (n = 31). Female players covered a total distance of 9556 ± 795 m and 103 ± 6 m × min^−1^ during matches [30–41, 43, 45, 48–63, 65–70]. Considering zones of intensity, women soccer players performed 1429 ± 702 m in MSR, 830 ± 1414 m in HSR and 267 ± 275 m in sprinting; other studies presented these variables relative to time (MSR = 15 ± 7 m × min^−1^; HSR = 4 ± 1 m × min^−1^; sprinting = 3 ± 2 m × min^−1^) [30, 33, 37, 47, 49, 55, 58, 66, 68–70]. Regarding the number of sprints, the players did a mean of 30 ± 19 sprint actions per match [34, 45, 53, 54, 58, 61, 67]. Moreover, studies [32–35, 41, 43, 46, 48, 54, 56, 63, 65, 67, 69, 70] reported that female players completed a total of 165 ± 129 ACC and 146 ± 141 DEC actions during the match. These actions were also presented in frequency per minute [30, 43, 48, 49, 54, 57, 66, 68], distance travelled [31, 36] and duration [38] (Table 2).

**TABLE 2:** Summary of the match demands in soccer.

Study (year)	Sport	Country	Players (n)	Age (years) Height (cm) Mass (kg)	Match (n)	Device	TD (m)	TD (m · min^−1^)	MSR (m) 12.6–19.8 km · h^−1^	HSR (m) 19.8–25.2 km · h^−1^	Sprint (m) ≥ 25.2 km · h^−1^	ACC (n)	DEC (n)
Andersson et al. (2010) [51]	Soccer	Sweden-Denmark	17	27 ± 1 168 ± 2 61 ± 1	6	TMA (Canon DM-MV 600, Canon Inc.)	9800 ± 141	N-R	N-R	1430 ± 141 (≥ 18 km · h^−1^)	239 ± 25 (≥ 25 km · h^−1^)	N-R	N-R

Bradley et al. (2014) [60]	Soccer	N-R	59	N-R	N-R	TMA (Multiple-camera system, Amisco Pro) 25 Hz	10754 ± 150	N-R	2374 ± 70 (12–18 km · h^−1^)	718 ± 34 (18–25 km · h^−1^)	N° 59 ± 9 (≥ 25 km · h^−1^)	N-R	N-R

Choi et al. (2022) [67]	Soccer	South Korea	24	29 ± 4 166 ± 5 59 ± 6	21	GPS (APEX STATSports) 10 Hz	9520 ± 676		1685 ± 395 (13–19 km · h^−1^)	371 ± 82 (19–23 km · h^−1^)	129 ± 81 Nº 16 ± 5 (≥ 23 km · h^−1^)	75 ± 13 (N-R)	85 ± 15 (N-R)

Datson et al. (2017) [47]	Soccer	N-R	107	N-R	1–4	TMA (Prozone Sports Ltd., Leeds)	10321 ± 859	N-R	2520 ± 580 (14–19.8 km · h^−1^)	776 ± 247 (≥ 19.8 km · h^−1^)	168 ± 82 (≥ 25 km · h^−1^)	N-R	N-R

Datson et al. (2019) [61]	Soccer	N-R	107	N-R	2–4	TMA (Semi-automated multi-camera image recognition system, STATS)	N-R	N-R	N-R	N° 169 ± 50 (≥ 19.8 km · h^−1^)	N° 33 ± 13 (≥ 25 km · h^−1^)	N-R	N-R

DeWitt et al. (2018) [45]	Soccer	USA	18	25 ± 3 168 ± 5 61 ± 5	20	GPS (Optimeye S5, Catapult Innovations) 10 Hz	8883 ± 877	99 ± 22	570 ± 407 (≥ 17.8 km · h^−1^)	N-R	N° 9 ± 11 (≥ 22.7 km · h^−1^)	N-R	N-R

Diaz-Seradilla et al. (2022) [58]	Soccer	Spain	17	23 ± 5 166 ± 6 60 ± 7	1	GPS (WIMU PRO, Real rack Systems) 10 Hz	9347 ± 1013	96 ± 9	1110 ± 332 12 ± 4 m · min^−1^ (≥ 16 km · h^−1^)	N-R	235 ± 21 N° 1 3 ± 4 3 ± 2 m · min^−1^ (≥ 21 km · h^−1^)	32 ± 2 m · min^−1^ (N-R)	32 ± 1 m · min^−1^ (N-R)

Fernandes et al. (2022) [56]	Soccer	Portugal	10	24 ± 2 165 ± 6 58 ± 9	15	GPS (PlayerTeck, Catapult) 10 Hz	7616 ± 395	90 ± 5	880 ± 102 (≥ 15 km · h^−1^)	N-R	N-R	177 ± 8 (2–3 m · s^−2^)	169 ± 5 (-2–3 m · s^−2^)

Gabbett et al. (2008) [62]	Soccer	Australia	30	21 ± 2 N-R N-R	12	TMA (37-mm digital video cameras, Sony, DCR-TRV 950E)	9967 ± 610 5618 ± 67 s	N-R	1484 ± 402 266 ± 71 s 15 ± 3% ^B^ (N-R)	N-R	995 ± 182 159 ± 35 s 10 ± 2% ^B^ (N-R)	N-R	N-R

García-Ceberino et al. (2022) [57]	Soccer	Spain	10	26 ± 4 166 ± 1 61 ± 7	3	GPS (SPRO, RealTrack Systems) 18 Hz	N-R	91 ± 12	2 ± 2 N° · min^−1^ (N-R)	N-R	7 ± 15 n · min^−1^ (N-R)	31 ± 3 n · min^−1^ (N-R)	32 ± 3 n · min^−1^ (N-R)

Gonçalves et al. (2021) [54]	Soccer	Portugal	22	25 ± 6 162 ± 7 59 ± 9	10	GPS (SPI HPU, GPSports) 15 Hz	8237 ± 206	100 ± 1	758 ± 50 9 ± 0.2 m · min^−1^ (14–18 km · h^−1^)	306 ± 46 4 ± 0.4 m · min^−1^ (18–24 km · h^−1^)	N° 15 ± 0.1 22 ± 3 (≥ 24 km · h^−1^)	41 ± 0.6 0.5 ± 0.02 n · min^−1^ (2–3 m · s^−2^)	44 ± 0.2 0.5 ± 0.03 n · min^−1^ (-2–3 m · s^−2^)

Griffin et al. (2021) [38]	Soccer	Australia	33	NAT = 15 26 ± 3 167 ± 8 61 ± 6 INT = 18 26 ± 4 167 ± 8 60 ± 7	36	GPS (SPI HPU, GPSports) 10 Hz	9080 ± 499	N-R	687 ± 112 (16–20 km · h^−1^)	335 ± 40 (≥ 20 km · h^−1^)	N-R	176 ± 17 s (2–3 m · s^−2^)	172 ± 13 s (2–3 m · s^−2^)

Hewitt et al. (2014) [50]	Soccer	Austalia	15	23 ± 1 170 ± 1 65 ± 1	13	GPS (MinimaxX v2.5, Catapult Innovations) 5 Hz	9631 ± 175	N-R	2407 ± 125 (12–19 km · h^−1^)	338 ± 30 (≥ 19 km · h^−1^)	N-R	N-R	N-R

Julian et al. (2021) [37]	Soccer	Germany	15	23 ± 4 169 ± 1 64 ± 8	4–7	GPS (Tracktics TT01) 5 Hz	N-R	103 ± 1	18 ± 2 m · min^−1^ (13–20 km · h^−1^)	4 ± 0.2 m · min^−1^ N° 24 ± 13 (≥ 20 km · h^−1^)	N-R	N-R	N-R

Kobal et al. (2022a) [69]	Soccer	Brazil	24	28 ± 5 164 ± 5 59 ± 8	38	GPS (Catapult Innovations) 10 Hz	9830 ± 42	104 ± 3	N-R	7234 ± 327 8 ± 0.4 m · min^−1^ (≥ 18 km · h^−1^)	N-R	726 ± 15 (≥ 3 m · s^−2^)	912 ± 2 (≥ 3 m · s^−2^)

Kobal et al. (2022b) [68]	Soccer	Brazil	23	28 ± 5 165 ± 5 59 ± 5	14	GPS (Catapult Innovations) 10 Hz	N-R	96 ± 14	N-R	8 ± 3 m · min^−1^ (≥ 18 km · h^−1^)	N-R	1 ± 0.2 n · min^−1^ (≥ 3 m · s^−2^)	1 ± 0.2 n · min^−1^ (≤ -3 m · s^−2^)

Krustrup et al. (2005) [53]	Soccer	Denmark	14	24 ± 8 167 ± 17 58 ± 22	4	TMA (VHS movie camera NV-M50, Panasonic)	10300 (9700–1300)	N-R	N-R	1310 (700–1700) 4.8% * (2.8–6.1) (≥ 18 km · h^−1^)	160 (50–280) N° 26 (9–43) (≥ 25 km · h^−1^)	N-R	N-R

Krustrup et al. (2021) [36]	Soccer	N-R	17	23 ± 4 166 ± 5 60 ± 7	1	GPS (S5, Catapult Innovations) 10 Hz	8500 ± 1200	N-R	903 ± 275 (16–20 km · h^−1^)	N-R	N-R	233 ± 52 m (≥ 2 m · s^−2^)	172 ± 40 m (≤ -2 m · s^−2^)

Mara et al. (2016) [63]	Soccer	Australia	12	24 ± 4 172 ± 5 65 ± 5	7	TMA (High-definition video cameras, Legria HF R38, Canon) 25 Hz	N-R	N-R	N-R	N-R	N-R	423 ± 126 (≥ 2 m · s^−2^)	430 ± 125 (≤ -2 m · s^−2^)

Mara et al. (2017) [46]	Soccer	Australia	12	24 ± 4 172 ± 5 65 ± 5	7	TMA (8 stationary high-definition video cameras Legria HF R38; Canon)	10025 ± 775	N-R	2452 ± 36 (12–19 km · h^−1^)	615 ± 258 N° 70 ± 29 (≥ 19 km · h^−1^)	N-R	N-R	N-R

Meylan et al. (2016) [49]	Soccer	N-R	13	27 ± 5 170 ± 6 66 ± 5	34	GPS (MinimaX S4, Catapult Innovations) 10 Hz	N-R	107 ± 16	6 ± 2 m · min^−1^ (16–20 km · h^−1^)	3 ± 1 m · min^−1^ (≥ 20 km · h^−1^)	N-R	2 ± 1 n · min^−1^ (≥ 2.26 m · s^−2^)	N-R

Mohr et al. (2008) [52]	Soccer	Sweden-Denmark	34	N-R	1–2	TMA (VHS movie cameras NV-M50)	10385 ± 150	N-R	N-R	1490 ± 95 (≥ 18 km · h^−1^)	420 ± 35 (≥ 25 km · h^−1^)	N-R	N-R

Nakamura et al. (2017) [64]	Soccer	Brazil	11	21 ± 3 164 ± 4 60 ± 8	10	GPS (SPI 119 Elite, GPSports Systems). 5 Hz	N-R	N-R	N-R	285 ± 164 N° 18 ± 9 3 ± 0.5 s (≥ 20 km · h^−1^)	N-R	N-R	N-R

Panduro et al. (2021) [34]	Soccer	Denmark	94	23 ± 4 170 ± 6 64 ± 6	2–4	GPS (Polar Team Pro Electro Oy) 10 Hz	10033 ± 454	N-R	1496 ± 256 (≥ 15 km · h^−1^)	676 ± 156 (≥ 18 km · h^−1^)	N° 49 ± 27 (≥ 25 km · h^−1^)	8 ± 5 (3–5 m · s^−2^)	15 ± 4 (-3–5 m · s^−2^)

Park et al. (2018) [42]	Soccer	N-R	27	25 ± 4 169 ± 5 63 ± 4	52	GPS (MinimaX S4, Catapult Innovations) 10 Hz	N-R	N-R	843 (812–876) (12–20 km · h^−1^)	101 (96–107) (≥ 20 km · h^−1^)	N-R	N-R	N-R

Principe et al. (2021) [35]	Soccer	Brazil	23	28 ± 5 165 ± 6 61 ± 5	22	GPS (Polar Team Pro Electro Oy) 10 Hz	8017 ± 360	N-R	2025 ± 224 (12–20 km · h^−1^)	306 ± 35 (≥ 20 km · h^−1^)	N-R	240 ± 18 (≥ 2 m · s^−2^)	242 ± 17 (≤ -2 m · s^−2^)

Ramos et al. (2017) [65]	Soccer	Brazil	12	18 ± 1 167 ± 6 62 ± 6	7	GPS (MinimaxX S5, Catapult Innovations) 10 Hz	8704 ± 432	N-R	688 ± 183 (16–20 km · h^−1^)	223 ± 120 (≥ 20 km · h^−1^)	N-R	15 ± 2 (≥ 2 m · s^−2^)	17 ± 6 (≤ -2 m · s^−2^)

Ramos et al. (2019a) [41]	Soccer	Brazil	17	27 ± 4 187 ± 5 61 ± 4	6	GPS (MinimaxX S5, Catapult Innovations) 10 Hz	10110 ± 245	N-R	736 ± 153 (16–20 km · h^−1^)	307 ± 80 (≥ 20 km · h^−1^)	N-R	214 ± 3 (≥ 1 m · s^−2^)	174 ± 4 (≤ -1 m · s^−2^)

Ramos et al. (2019b) [66]	Soccer	Brazil	21	26 ± 4 167 ± 6 N-R	6	GPS (MinimaxX S5, Catapult Innovations) 10 Hz	N-R	109 ± 4	22 ± 2 m · min^−1^ (12–20 km · h^−1^)	3 ± 1 m · min^−1^ (≥ 20 km · h^−1^)	N-R	0.05 ± 0.01 n · min^−1^ (≥ 2.5 m · s^−2^)	0.12 ± 0.03 n · min^−1^ (≤ -2.5 m · s^−2^)

Romero-Moraleda et al. (2021) [33]	Soccer	Spain	18	26 ± 6 164 ± 5 59 ± 6	94	GPS (SPI Pro X, GPSports Systems) 5 Hz	9040 ± 938	95 ± 9	1108 ± 294 12 ± 2 m · min^−1^ (≥ 15 km · h^−1^)	N-R	N-R	255 ± 40 (≥ 1 m · s^−2^)	78 ± 16 (≤ -1 m · s^−2^)

Scott et al. (2020a) [39]	Soccer	USA	36	24 ± 4 168 ± 6 63 ± 5	220	GPS (Optimeye S5, Catapult Innovations) 10 Hz	10068 ± 615	N-R	2401 ± 454 (≥ 12.5 km · h^−1^)	398 ± 153 (≥ 19 km · h^−1^)	162 ± 69 (≥ 22.5 km · h^−1^)	N-R	N-R

Scott et al. (2020b) [40]	Soccer	USA	220	25 ± 3 167 ± 6 64 ± 6	N-R	GPS (Optimeye S5, Catapult Innovations) 10 Hz	10073 ± 425	N-R	2409 ± 263 (≥ 12.5 km · h^−1^)	479 ± 114 (≥ 19 km · h^−1^)	139 ± 32 (≥ 22.5 km · h^−1^)	N-R	N-R

Trewin et al. (2017) [43]	Soccer	N-R	45	N-R	7 ± 6	GPS (MinimaX S4, Catapult Innovations) 10 Hz	10368 ± 952	108 ± 10	930 ± 348 10 ± 4 m · min^−1^ (≥ 16 km · h^−1^)	0.2 ± 0.1 N° · min^−1^ N° 20 ± 9 (≥ 20 km · h^−1^)	N-R	174 ± 33 1.8 ± 0.3 n · min^−1^ (≥ 2 m · s^−2^)	N-R

Trewin et al. (2018) [48]	Soccer	N-R	45	24 ± 13 N-R N-R	47	GPS (MinimaX S4, Catapult Innovations) 10 Hz	N-R	107 ± 10	10 ± 3 m · min^−1^ (≥ 16 km · h^−1^)	0.2 ± 0.1 N° · min^−1^ N° 20 ± 9 (≥ 20 km · h^−1^)	N-R	1.8 ± 0.3 n · min^−1^ (≥ 2 m · s^−2^)	N-R

Vescovi et al. (2012) [44]	Soccer	USA	71	N-R	12	GPS (SPI Pro, GPSports) 5 Hz	N-R	N-R	N-R	550 ± 186 (18–21 km · h^−1^)	N-R	N-R	N-R

Vescovi et al. (2019) [55]	Soccer	N-R	28	N-R	2	GPS (SPI Pro, GPSports) 5 Hz	N-R	111 ± 12	27 ± 1 m · min^−1^ (12–20 km · h^−1^)	4 ± 1 m · min^−1^ (≥ 20 km · h^−1^)	N-R	N-R	N-R

Villaseca-Vicuña et al. (2021) [32]	Soccer	Chile	26	27 ± 3 158 ± 21 59 ± 5	26	GPS (Optimeye S5, Catapult Innovations) 10 Hz	9415 ± 766	108 ± 7	N-R	515 ± 162 N° 35 ± 11 (≥ 18 km · h^−1^)	N-R	102 ± 28 (≥ 2 m · s^−2^)	N-R

Villaseca-Vicuña et al. (2023) [70]	Soccer	Chile	10	27 ± 3 163 ± 4 60 ± 5	6	GPS (Optimeye S5, Catapult Innovations) 10 Hz	9737 ± 448	108 ± 4	N-R	566 ± 49 6 ± 1 m · min^−1^ Nº 42 ± 4 (≥ 18 km · h^−1^)	N-R	N-R	N-R

Winther et al. (2021) [31]	Soccer	Norway	108	22 ± 4 N-R N-R	60	GPS (APEX STATSports) 10 Hz	9603 ± 480	N-R	1499 ± 300 (≥ 16 km · h^−1^)	369 ± 116 (≥ 20 km · h^−1^)	N-R	486 ± 62 m (≥ 2 m · s^−2^)	389 ± 69 m (≤ -2 m · s^−2^)

Yousefian et al. (2021) [30]	Soccer	Sweden	21	27 ± 4 172 ± 5 65 ± 4	7	GPS (S5, Catapult Innovation) 10 Hz	N-R	99 ± 4	22 ± 3 m · min^−1^ (12–19 km · h^−1^)	4 ± 0.5 m · min^−1^ (≥ 19 km · h^−1^)	N-R	0.2 ± 0.04 n · min^−1^	0.2 ± 0.04 n · min^−1^

* Percentage of total time.

B Percentage of total distance. ACC: accelerations; DEC: decelerations; GPS: global positions system; HSR: high-speed running; MSR: moderate-speed running; N-R: no reported; TD: total distance; TMA: time-motion analysis; USA: United States of America.

### Rugby

Table 3 depicts the match demands, anthropometric data and origin of rugby, rugby sevens, and field hockey. Out of the 23 results found in rugby, 6 correspond to rugby union [71, 73, 74, 76, 92, 93], 3 to rugby league [72, 75, 87] and 14 to rugby sevens [76–82, 84–86, 88–91]. Regarding external match load, GPS devices were used. Players covered an average of 5351 ± 855 m, while only three studies reported the density of 70 ± 8 m × min^−1^ [71–73]. Rugby female players performed 916 ± 386 m in MSR and a mean of 135 ± 63 m HSR per match. Few studies presented the locomotive zones of intensity in terms of proportion (18 ± 9%) [73, 76] and density (21 ± 4 m × min ^−1^) [75]. The authors reported that players did a mean of 8 ± 8 sprints per game [75]. Regarding ACC, one study [76] reported number (Nº = 19 ± 8) and two [71, 72] the frequency per minute (0.7 ± 0.4 ACC × min^−1^) (Table 3).

**TABLE 3:** Summary of the match demands of rugby union and sevens and field hockey.

Study (year)	Sport	Country	Players (n)	Age (years) Height (cm) Mass (kg)	Match (n)	Device	TD (m)	TD (m · min^−1^)	MSR (m) 12.6–19.8 km · h^−1^	HSR (m) 19.8–25.2 km · h^−1^	Sprint (m) ≥ 25.2 km · h^−1^	ACC (n)	DEC (n)
Busbridge et al. (2020) [92]	Rugby Union	New Zealand	20	24 ± 4 170 ± 6 79 ± 11	7	GPS (VX Log 340b, Firmware V1.62-03, VX Sport) 10 Hz	5812 ± 470	N-R	483 ± 276 7 ± 4 m · min^−1^ (≥ 16 km · h^−1^)	N-R	N-R	N-R	N-R

Callanan et al. (2021) [73]	Rugby Union	Ireland	128	Forwards 26 ± 4 172 ± 7 80 ± 8 Backs 25 ± 4 167 ± 5 70 ± 6	12	Triaxial magnetometer (PlayerTek, Catapult Innovations) 10 Hz	5696 ± 822	68 ± 7	1380 ± 383 24%* (10–18 km · h^−1^)	220 ± 156 4%* (≥ 18 km · h^−1^)	N-R	N-R	N-R

Nolan et al. (2023) [93]	Rugby Union	N-R	53	N-R	12	GPS (STATSports Apex; STATSports) 10 Hz	4177 ± 206	60 ± 9	1254 ± 637 18 ± 6 m · min^−1^ (10–19.5 km · h^−1^)	106 ± 126 1 ± 2 m · min^−1^ (≥ 19.5 km · h^−1^)	N-R	N-R	N-R

Sheppy et al. (2020) [74]	Rugby Union	Wales	29	24 ± 3 167 ± 1 75 ± 11	8	GPS (Optimeye S5, Catapult Innovations) 10 Hz	5784 ± 569	N-R	N-R	N-R	N-R	N-R	N-R

Suarez-Arrones et al. (2014) [76]	Rugby Union	Spain	8	Backs = 4 27 ± 3 170 ± 2 68 ± 4 Forwards = 4 27 ± 2 174 ± 6 77 ± 10	1	GPS (SPI Pro X; GPSports) 5 Hz	5820 ± 512	N-R	658 ± 264 11.3%^*^ (14–20 km · h^−1^)	73 ± 107 N° 5 ± 5 1.2%^*^ (≥ 20 km · h^−1^)	N-R	19 ± 8 (≥ 3 m · s^−2^)	N-R

Woodhouse et al. (2021) [71]	Rugby Union	England	78	25 ± 4 171 ± 6 77 ± 10	53	GPS (Viper, STATSports) 18 Hz	4271 ± 814	66 ± 4	1314 ± 367 21 ± 4 m · min^−1^ (11–20 km · h^−1^)	N° 8 ± 5 0.1 ± 0.07 N · min^−1^ (≥ 20 km · h^−1^)	N-R	1 ± 0.1 n · min^−1^ (2–3 m · s^−2^)	1 ± 0.1 n · min^−1^ (-2 to -3 m · s^−2^)

Emmonds et al. (2020) [75]	Rugby League	N-R	58	N-R	9	GPS (Optimeye S5, Catapult Innovations) 10 Hz	5383 ± 780	75 ± 2	N-R	140 ± 90 2 ± 1 m · min^−1^ (≥ 18 km · h^−1^)	N° 8 ± 8 0.1 ± 0.1 m · min^−1^ (≥ 25 km · h^−1^)	N-R	N-R

Quinn et al. (2019) [87]	Rugby League	Australia	18	26 ± 4 N-R N-R	7	GPS (SPI Pro X, GPSports) 10 Hz	6712 (6203–6951)	N-R	542 (368–644) (≥ 15 km · h^−1^)	N-R	N-R	N-R	N-R

Newans et al. (2021) [72]	Rugby League	Australia	117	26 ± 5 170 ± 1 77 ± 12	4 ± 2	GPS (Optimeye S5, Catapult Innovations) 10 Hz	4504 ± 1029	79 ± 2	774 ± 210 (≥ 12 km · h^−1^)	N-R	N-R	0.4 ± 0.02 n · min^−1^ (N-R)	N-R

Clarke et al. (2014a) [88]	Rugby sevens	Australia	12	25 ± 5 168 ± 1 69 ± 7	N-R	GPS (SPI Pro X; GPSports) 5 Hz	N-R	86 ± 7	N-R	N-R	N-R	N-R	N-R

Clarke et al. (2014b) [86]	Rugby sevens	Australia	12	23 ± 5 168 ± 1 68 ± 8	6	GPS (SPI HPU, GPSports) 5 Hz	1164 ± 255	106 ± 7	36 ± 2%^*^ (≥ 12.6 km · h^−1^)	13 ± 2%^*^ (≥ 18 km · h^−1^)	N-R	N-R	N-R

Clarke et al. (2015) [85]	Rugby sevens	Australia	12	22 ± 2 167 ± 4 66 ± 5	4–6	GPS (SPI HPU, GPSports) 5 Hz	3142 ± 879	95 ± 10	629 19%* (12–18 km · h^−1^)	482 ± 14 13%* (≥ 18 km · h^−1^)	N-R	N-R	N-R

Clarke et al. (2017) [90]	Rugby sevens	Australia	11	(N-R) 169 ± 2 69 ± 4	12	GPS (SPI HPU, GPSports) 5 Hz	1078 ± 197	86 ± 4	323 ± 87 30 ± 4%^*^ (12–18 km · h^−1^)	120 ± 41 11 ± 3%* (≥ 18 km · h^−1^)	149 ± 39 14 ± 3%* (N-R)	N-R	N-R

Conte et al. (2022) [77]	Rugby sevens	Brazil	14	Backs = 6 24 ± 3 161 ± 7 59 ± 5 Forwards = 8 22 ± 3 167 ± 5 71 ± 6	12	GPS (OptimEye X4, Catapult Innovations) 10 Hz	1119 ± 416	92 ± 1	66 ± 4 5 ± 0.2 m · min^−1^ (18–20 km · h^−1^)	97 ± 25 8 ± 2 m · min^−1^ (≥ 20 km · h^−1^)	N-R	14 ± 2 1 ± 0.1 n · min^−1^ (≥ 1.8 m · s^−2^)	21 ± 1 1 ± 0.4 n · min^−1^ (≤ -1.8 m · s^−2^)

Del coso et al. (2013) [89]	Rugby sevens	Spain-Netherlands	8	23 ± 2 166 ± 7 66 ± 7	3	GPS (SPI Pro X, GPSports) 5 Hz	N-R	87 ± 8	N-R	N-R	N-R	N-R	N-R

Doeven et al. (2019) [91]	Rugby sevens	N-R	10	25 ± 4 169 ± 4 64 ± 5	5	GPS (JOHAN Sports) 10 Hz	1466 ± 120	N-R	366 ± 45 (≥ 12 km · h^−1^)	N-R	N-R	N-R	N-R

Goodale et al. (2006) [84]	Rugby sevens	N-R	20	24 ± 4 168 ± 6 69 ± 5	N-R	GPS (Minimax S4, Catapult Innovations) 10 Hz	1352 ± 306	87 ± 11	255 ± 94 16 ± 5 m · min^−1^ (12–18 km · h^−1^)	112 ± 51 7 ± 3 m · min^−1^ (18–23 km · h^−1^)	38 ± 31 2 ± 2 m · min^−1^ (≥ 23 km · h^−1^)	N-R	N-R

Malone et al. (2020) [78]	Rugby sevens	N-R	27	24 ± 2 168 ± 7 68 ± 4	36	GPS (Viper; STATSports) 10 Hz	1625 ± 132	116 ± 9	N-R	199 ± 44 14 ± 3 m · min^−1^ (16–20 km · h^−1^)	118 ± 45 N° 3.5 ± 1 (≥ 20 km · h^−1^)	2 ± 1 (≥ 2.5 m · s^−2^)	N-R

Misseldine et al. (2018) [79]	Rugby sevens	N-R	12	Fowards = 5 27 ± 2 170 ± 3 70 ± 2 Backs = 7 24 ± 5 167 ± 5 62 ± 4	6	GPS (JOHAN trackers, JOHAN Sports) 5 Hz	1564 ± 52	98 ± 1	255 ± 30 17 ± 1%* (14–20 km · h^−1^)	86 ± 37 N° 6 ± 1 6 ± 3%* (≥ 20 km · h^−1^)	N-R	N-R	N-R

Portillo et al. (2014) [82]	Rugby sevens	Spain	20	INT = 10 26 ± 4 167 ± 7 65 ± 5 NAT = 10 32 ± 6 167 ± 3 66 ± 5	4	GPS (SPI HPU, GPSports) 5 Hz	1503 ± 197	N-R	312 ± 94 (14–20 km · h^−1^)	83 ± 51 N° 4 ± 3 (≥ 20 km · h^−1^)	N-R	4 ± 1 (≥ 2 m · s^−2^)	N-R

Reyneke et al. (2018) [80]	Rugby sevens	N-R	15	24 ± 4 168 ± 7 67 ± 6	15	GPS (VX sport 220,Visuallex Sport International) 4 Hz	N-R	90 ± 3	18 ± 1 m · min^−1^ (12–18 km · h^−1^)	6 ± 3 m · min^−1^ (18–21 km · h^−1^)	4 ± 1 m · min^−1^ (≥ 21 km · h^−1^)	N-R	N-R

Suarez-Arrones et al. (2012) [83]	Rugby sevens	N-R	12	28 ± 4 165 ± 6. 64 ± 5	5	GPS (SPI Elite, GPSports) 1 Hz	1556 ± 189	N-R	437 ± 149 28%^*^ (12–20 km · h^−1^)	84 ± 65 5.4%* (≥ 20 km · h^−1^)	N-R	N-R	N-R

Vescovi et al. (2015) [81]	Rugby sevens	Canada	16	N-R	5	GPS (SPI Pro 5, GPSports) 5 Hz	1468 ± 88	95 ± 5	552 ± 76 36 ± 5 m · min^−1^ (8–16 km · h^−1^)	224 ± 55 14 ± 3 m · min^−1^ (16–20 km · h^−1^)	128 ± 67 8 ± 4 m · min^−1^ (20–32 km · h^−1^)	N-R	N-R

Choi et al. (2020) [96]	Field hockey	Korea	52	26 ± 3 165 ± 4 59 ± 5	65	GPS (SPI-HPU, GPSports) 15 Hz	5760 ± 88	N-R	859 ± 90 (≥ 15 km · h^−1^)	N-R	N-R	16 ± 1 (≥ 3 m · s^−2^)	32 ± 2 (≤ -3 m · s^−2^)

Delves et al. (2021) [101]	Field hockey	Australia	11	22 ± 2 167 ± 6 62 ± 7	14	GPS Catapult (OptimEye X4, Catapult Innovations) 10 Hz	5310 ± 50	N-R	N-R	325 ± 109 (≥ 18 km · h^−1^)	N-R	N-R	N-R

Kapteijns et al. (2021) [94]	Field hockey	N-R	20	23 ± 4 169 ± 5 62 ± 5	26	GPS (APEX, County Down, STATSports) 18 Hz	5384 ± 835	147 ± 16	796 ± 221 (15–19 km · h^−1^)	274 ± 105 (≥ 19 km · h^−1^)	N-R	27 ± 12 (≥ 3 m · s^−2^)	40 ± 15 (≤ -3 m · s^−2^)

Kim et al. (2016) [99]	Field hockey	N-R	32	28 ± 3 165 ± 4 60 ± 4	N-R	GPS (SPI-HPU, GPSports) 5 Hz	5268 ± 77	N-R	580 ± 11 (12–14 km · h^−1^)	775 ± 19 (18–24 km · h^−1^)	371 ± 9 n: 28 ± 1 (≥ 24 km · h^−1^)	N-R	N-R

McGuinness et al. (2018) [100]	Field hockey	N-R	16	23 ± 3 163 ± 13 66 ± 6	7	GPS (S5, Catapult Innovations) 10 Hz	5147 ± 628	113 ± 9	16 ± 5 m · min^−1^ (≥ 16 km · h^−1^)	N-R	N-R	N-R	N-R

McMahon et al. (2019) [98]	Field hockey	Ireland	19	23 ± 4 (N-R) 64 ± 6	13	GPS Catapult (OptimEye S5, Catapult Innovations) 10 Hz	5167 ± 1030	N-R	959 ± 294 298 ± 7 m · min^−1^ (11–19 km · h^−1^)	N-R	N-R	N-R	N-R

Morencos et al. (2019) [97]	Field hockey	Spain	16	25 ± 3 165 ± 5 58 ± 6	5	GPS (SPI ELITE, GPSport) 10 Hz	5834 ± 931	N-R	892 ± 41 (≥ 15 km · h^−1^)	848 ± 45 N° 65 ± 1 (≥ 21 km · h^−1^)	N-R	35 ± 5 3 ± 0.5 n · min^−1^ (2–3 m · s^−2^)	24 ± 3 2 ± 1 n · min^−1^ (2–3 m · s^−2^)

Sánchez-Migallón et al. (2020) [95]	Field hockey	N-R	30	23 ± 4 160 ± 1 60 ± 7	1	GPS (RealTrack Systems, WimuProTM) 10 Hz	5456 ± 699	N-R	852 ± 282 16 ± 5%* (12–18 km · h^−1^)	108 ± 76 1.98 ± 1.40%* (18–21 km · h^−1^)	n: 24 ± 29 0.5 ± 0.5%* (21–24 km · h^−1^)	N-R	N-R

^B^Percentage of total distance. ACC: accelerations; DEC: decelerations; GPS: global positions system; HSR: high-speed running; MSR: moderate-speed running; N-R: no reported; TD: total distance; TMA: time-motion analysis.

All rugby sevens studies used GPS devices to record external match load. Rugby sevens female players covered an average of 1549 ± 562 m and 94 ± 9 m × min^−1^. Regarding zones of intensity, female players performed 355 ± 168, 165 ± 129 and 108 ± 49 m in MSR, HSR, and sprinting respectively. Some studies [79, 83, 85, 86, 90] reported MSR and HSR in terms of proportion of TD (MSR = 28 ± 8%; HSR = 10 ± 4%; sprinting = 14 ± 3%). Regarding distance relative to time in MSR, HSR, and sprinting, players performed 19 ± 13, 10 ± 4, and 5 ± 3 m × min^−1^, respectively [77, 80, 81, 84]. Lastly, players performed 5 ± 1 sprints, 7 ± 6 ACC and 21 ± 1 DEC per match [77, 78, 82] (Table 3).

### Field hockey

In field hockey, GPS devices were used to record external match load (Table 3). Female players covered an average of 5433 ± 265 m TD [94–99, 101, 117], while only two studies reported this variable relative to time 130 ± 24 m × min^−1^ [94, 100]. Players covered 823 ± 131 m in MSR [94–99], 466 ± 326 m in HSR [94, 95, 97, 99, 101] and 371 ± 9 m in sprinting [99]. Moreover, female field hockey players performed 39 ± 23 sprints, 26 ± 10 ACC and 32 ± 8 DEC per game (Table 3).

### Basketball

Table 4 shows the results for basketball, handball and futsal. In basketball, the variables were recorded by TMA (n = 4) and LPS (n = 1). Female basketball players covered 5285 ± 2480 m per match (MSR = 459 ± 70 m; HSR = 1850 ± 12 m; sprint = 925 ± 184 m) [106, 109]. The minutes played were 27 ± 2 min, of which 16 ± 14%, 7 ± 4% and 7 ± 5% corresponded to MSR, HSR, and sprinting respectively [102–105, 107]. This metric was also reported in numbers of actions relative of time (MSR = 2 ± 0.6 m × min^−1^; HSR = 0.2 ± 0.5 m × min^−1^; sprint = 0.6 ± 0.6 m × m in^−1^) [104, 108].

**TABLE 4:** Summary of the match demands of basketball, handball, and futsal.

Study (year)	Sport	Country	Players (n)	Age (years) Height (cm) Mass (kg)	Match (n)	Device	TD (m)	Pload (AU · min^−1^)	MSR (m) 12.6–19.8 km · h^−1^	HSR (m) 19.8–25.2 km · h^−1^	Sprint (m) ≥ 25.2 km · h^−1^	ACC (n)	DEC (n)
Conte et al. (2015) [105]	Basketball	Italy	12	27 ± 4 184 ± 1 77 ± 15	5	TMA (Dartfish 6.0 hfixed camera, Sony HD AVCHD HDR-CX115)	N-R	N-R	N° 56 ± 16 9.6 ± 2.5%^*^ (N-R)	N°63 ± 16 11 ± 1.8%^*^ (N-R)	n: 44 ± 15 7.8 ± 2.2%^*^ (N-R)	N-R	N-R

Delextrat et al. (2012) [108]	Basketball	England	9	24 ± 4 173 ± 8 65 ± 11	1	TMA (JVC-x400)	N-R	N-R	N-R	N° 40 ± 14 02 ± 0.5 N° × min^−1^ (N-R)	n: 26 ± 16 1 ± 0.5 N° × min^−1^ (N-R)	N-R	N-R

Delextrat et al. (2017) [104]	Basketball	Spain	42	26 ± 4 183 ± 9 (N-R)	3	TMA (LINCE multiplatform sport analysis software Observesport) 25 Hz	N-R	N-R	1.2 ± 0.6 n × min^−1^ 4.9 ± 2.6%^*^ (≥ 9 km · h^−1^)	N-R	0.2 ± 0.2 n × min^−1^ 0.6 ± 0.6%^*^	N-R	N-R

Palmer et al. (2021) [102]	Basketball	Australia	12	25 ± 6 180 ± 11 79 ± 17	20	Triaxial accelerometer (GT9X Actigraph) 100 Hz	N-R	N-R	16.7%^*^ (15.7–17.4) (≥ 40–90% VO^2^)^◊^	3.3%^*^ (1.1–3.8) (90–100% VO^2^)^◊^	3.8%^*^ (2.5–5.3) (≥ 100% VO^2^)^◊^	N-R	N-R

Palmer et al. (2022) [107]	Basketball	Australia	13	25 ± 6 181 ± 11 79 ± 17	21	Triaxial accelerometer (GT9X Actigraph) 100 Hz	N-R	N-R	40.2%^*^ (35.9–49.1) (40–90% VO^2^ reserve)^◊^	10.7%^*^ (9.8–12.0) (90–100% VO^2^ reserve)^◊^	15.1%^*^ (9.7–25.0) (≥ 100% VO^2^ reserve)^◊^	N-R	N-R

Reina et al. (2022) [109]	Basketball	Spain	10	24 ± 3 195 ± 1 93 ± 16	1	LPS (WIMU PROTM systems RealTrack Systems)	3531 ± 310 69 ± 3 m × min^−1^	1 ± 0.15	459 ± 70 9 ± 1 m × min^−1^ (≥ 15 km · h^−1^)	N-R	N-R	18 ± 1 n × min^−1^	18 ± 1 n × min^−1^

Scanlan et al. (2012) [106]	Basketball	Australia	12	22 ± 4 174 ± 7 73 ± 14	1	TMA (Labviewsoftware, National Instruments) 7.5 Hz	7039 ± 446	N-R	N-R	1850 ± 13 (11 –25 km · h^−1^)	925 ± 184 (≥ 25 km · h^−1^)	N-R	N-R

Stauton et al. (2018) [103]	Basketball	Australia	10	27 ± 5 182 ± 8 81 ± 12	18	Triaxial accelerometer (Link; Actigraph) 100 Hz	N-R	N-R	11 ± 0.5%^*^ (60–90% VO^2^)^◊^	4 ± 1%^*^ (90–100% VO^2^)^◊^	6 ± 5%^*^ (100% VO^2^)^◊^	N-R	N-R

Kniubaite et al. (2019) [110]	Handball	Lithuania	8	23 ± 2 173 ± 5 68 ± 7	14	Triaxial accelerometer (IMU; Optimeye S5 Catapult Innovations) 100 Hz	N-R	9	N-R	N-R	N-R	N-R	N-R

Luteberget et al. (2016) [111]	Handball	Norway	20	25 ± 4 175 ± 4	9	Triaxial accelerometer (IMU; Optimeye S5 Catapult Innovations) 100 Hz	N-R	8.8 ± 2.1	N-R	N-R	N-R	0.7 ± 0.4 n × min^−1^ (≥ 2.5 m × s^−2^)	2.3 ± 0.9 n × min^−1^ (≤ -2.5 m × s^−2^)

Luteberget et al. (2017) [112]	Handball	Norway	31	22 ± 3 171 ± 6 68 ± 7	9	Triaxial accelerometer (IMU; Optimeye S5 Catapult Innovations) 100 Hz	N-R	9.85 ± 0.36	N-R	N-R	N-R	N-R	N-R

Manchado et al. (2013) [115]	Handball	Germany-Norway	25	25 ± 3 175 ± 6 68 ± 5	N-R	TMA (camera 25 Hz)	2882 ± 1506	N-R	752 ± 484 m 29.7 ± 3.9%^B^ 3.4 ± 0.6 n × min^−1^ (11–20 km · h^−1^)	272 ± 224 m 10.5 ± 4.1%^B^ 0.8 ± 0.4 n × min^−1^ (≥ 20 km · h^−1^)	N-R	16.7 ± 6.7 n × min^−1^ (1.5–3 m × s^−2^)	N-R

Michalsik et al. (2014) [114]	Handball	Denmark	24	26 ± 4 174 ± 6 70 ± 7	1–8	TMA (No reported)	4002 ± 551	N-R	93 ± 67 m 0.8 ± 0.5% ^*^ 2.5 ± 1.8% ^B^ (≥ 15.5 km · h^−1^)	10 ± 11 m 0.1%^*^ 0.2% ^B^ (≥ 22 km · h^−1^)	N-R	N-R	N-R

Wik et al. (2016) [113]	Handball	Norway	18	25 ± 4	9	Triaxial accelerometer (IMU; Optimeye S5 Catapult Innovations) 100 Hz	N-R	9.5 ± 1.1	N-R	N-R	N-R	N-R	N-R

Oliva Lozano et al. (2021) [116]	Futsal	Spain	14	24 ± 4 165 ± 6 63 ± 6	5	LPS (WIMU PROTM systems RealTrack Systems) 33 Hz	N-R	N-R	N-R	5 ± 0.4 m · min (≥ 20 km · h^−1^)	N-R	0.4 ± 0.3 m × min^−1^ (4–5 m × s−2) 28 ± 0.2 m × min^−1^ 240 ± 55 m × min^−1^	28 ± 0.2 m × min^−1^

* Percentage of total time;

B Percentage of total distance. ACC: accelerations; DEC: decelerations; GPS: global positions system; HSR: high-speed running; MSR: moderate-speed running; N-R: no reported; TD: total distance; TMA: time-motion analysis.

### Handball

In handball, variables were extracted using IMU (n = 4) and TMA (n = 2) (Table 4). Handball female players competed an average of 37.6 ± 11.2 min [111, 113, 114] and covered 3442 ± 792 m TD [114, 115] during match-play. Regarding the intensity of the matches (Pload), three studies reported that a mean of 9 ± 0.5 au · min^−1^ was performed [110–113]. Female handball players covered 423 ± 466 m in MSR and 141 ± 185 m in sprinting, which correspond to 16 ± 19% and 5 ± 7 % respectively of TD [114, 115]. During the competition, the players performed 8.7 ± 11 ACC × min^−1^ and 2.3 ± 0.9 DEC × min^−1^ [111, 115] (Table 4).

### Futsal

In futsal, only one study met the inclusion criteria [116]. Five matches were monitored using LPS. The players covered a mean of 5 ± 0.4 m × min^−1^ in HSR. The maximum ACC was 6 ± 0.2 m × s^−2^; a total of 240 ± 55 m × min^−1^ in ACC was recorded, with a total of 28 ± 0.3 ACC × min^−1^, of which 0.4 ± 0.3 ACC × min^−1^ was performed above 4–5 m × s^−2^. The maximum DEC was 6 ± 2 m × s^−2^ and an average of 28 ± 0.2 per minute [116] (Table 4).

**TABLE:** Preferred Reporting Items for Systematic reviews and Meta-Analyses extension for Scoping Reviews (PRISMA-ScR) Checklist

SECTION	ITEM	PRISMA-ScR CHECKLIST ITEM	REPORTED ON PAGE #
**TITLE**

Title	1	Identify the report as a scoping review.	175

**ABSTRACT**			

Structured summary	2	Provide a structured summary that includes (as applicable): background, objectives, eligibility criteria, sources of evidence, charting methods, results, and conclusions that relate to the review questions and objectives.	175

**INTRODUCTION**			

Rationale	3	Describe the rationale for the review in the context of what is already known. Explain why the review questions/objectives lend themselves to a scoping review approach.	175–176

Objectives	4	Provide an explicit statement of the questions and objectives being addressed with reference to their key elements (e.g., population or participants, concepts, and context) or other relevant key elements used to conceptualize the review questions and/or objectives.	176

**METHODS**			

Protocol and registration	5	Indicate whether a review protocol exists; state if and where it can be accessed (e.g., a Web address); and if available, provide registration information, including the registration number.	176

Eligibility criteria	6	Specify characteristics of the sources of evidence used as eligibility criteria (e.g., years considered, language, and publication status), and provide a rationale.	176

Information sources*	7	Describe all information sources in the search (e.g., databases with dates of coverage and contact with authors to identify additional sources), as well as the date the most recent search was executed.	176

Search	8	Present the full electronic search strategy for at least 1 database, including any limits used, such that it could be repeated.	176

Selection of sources of evidence†	9	State the process for selecting sources of evidence (i.e., screening and eligibility) included in the scoping review.	176

Data charting process‡	10	Describe the methods of charting data from the included sources of evidence (e.g., calibrated forms or forms that have been tested by the team before their use, and whether data charting was done independently or in duplicate) and any processes for obtaining and confirming data from investigators.	176

Data items	11	List and define all variables for which data were sought and any assumptions and simplifications made.	176

Critical appraisal of individual sources of evidence§	12	If done, provide a rationale for conducting a critical appraisal of included sources of evidence; describe the methods used and how this information was used in any data synthesis (if appropriate).	176

Synthesis of results	13	Describe the methods of handling and summarizing the data that were charted.	176

**RESULTS**			

Selection of sources of evidence	14	Give numbers of sources of evidence screened, assessed for eligibility, and included in the review, with reasons for exclusions at each stage, ideally using a flow diagram.	176–178

Characteristics of sources of evidence	15	For each source of evidence, present characteristics for which data were charted and provide the citations.	176–178

Critical appraisal within sources of evidence	16	If done, present data on critical appraisal of included sources of evidence (see item 12).	178


Results of individual sources of evidence	17	For each included source of evidence, present the relevant data that were charted that relate to the review questions and objectives.	176–194

Synthesis of results	18	Summarize and/or present the charting results as they relate to the review questions and objectives.	176–194

**DISCUSSION**			

Summary of evidence	19	Summarize the main results (including an overview of concepts, themes, and types of evidence available), link to the review questions and objectives, and consider the relevance to key groups.	193–194

Limitations	20	Discuss the limitations of the scoping review process.	194

Conclusions	21	Provide a general interpretation of the results with respect to the review questions and objectives, as well as potential implications and/or next steps.	194–195

**FUNDING**			

Funding	22	Describe sources of funding for the included sources of evidence, as well as sources of funding for the scoping review. Describe the role of the funders of the scoping review.	195

Note: JBI = Joanna Briggs Institute; PRISMA-ScR = Preferred Reporting Items for Systematic reviews and Meta-Analyses extension for Scoping Reviews.

* Where *sources of evidence* (see second footnote) are compiled from, such as bibliographic databases, social media platforms, and Web sites.

† A more inclusive/heterogeneous term used to account for the different types of evidence or data sources (e.g., quantitative and/or qualitative research, expert opinion, and policy documents) that may be eligible in a scoping review as opposed to only studies. This is not to be confused with *information sources* (see first footnote).

‡ The frameworks by Arksey and O’Malley (6) and Levac and colleagues (7) and the JBI guidance (4, 5) refer to the process of data extraction in a scoping review as data charting.

§ The process of systematically examining research evidence to assess its validity, results, and relevance before using it to inform a decision. This term is used for items 12 and 19 instead of “risk of bias” (which is more applicable to systematic reviews of interventions) to include and acknowledge the various sources of evidence that may be used in a scoping review (e.g., quantitative and/or qualitative research, expert opinion, and policy document).

*From:* Tricco AC, Lillie E, Zarin W, O’Brien KK, Colquhoun H, Levac D, et al. PRISMA Extension for Scoping Reviews (PRISMAScR): Checklist and Explanation. Ann Intern Med. 2018;169:467–473. doi: 10.7326/M18-0850.

## DISCUSSION

This scoping review provides an overview of research on the physical demands of female athletes in elite team sports. Football was the most researched sport. In contrast, women’s indoor sports have been less researched. In particular, GPS have emerged as the main devices used to monitor the physical demands of outdoor team sports (i.e., soccer, rugby, field hockey) and, on the other hand, accelerometers and TMA have been more commonly used to measure the physical demands of indoor sports (i.e., basketball, handball, futsal). It should be noted that the demands of matches vary significantly between sports, as each sport has its own characteristics and requirements. Therefore, a thorough understanding of the physical demands of different team sports is crucial to optimise training and performance, reduce the risk of injury and improve player well-being.

Considering female soccer, TD covered were ~9556 m and 103 ± 6 m × min^−1^ when considered in relative distance. Similar results were obtained in a previous meta-analysis [118] but with male players. Regarding intensity zones, there was observed high variability in MSR (range: 570–2520 m), HSR (range: 101–1490 m), and sprinting (range: 22–995 m). This could be explained by the differences in devices (TMA vs. GPS), sampling frequencies (i.e. 1–15 Hz) or ranges of velocity used. The same was observed when relative distance in MSR (6–27 m × min^−1^) was analysed. Although the number of sprints was reported, no previous consensus was established about the velocity that should be considered (e.g. > 21 km × h^−1^ – > 25 km × h^−1^); this phenomenon could explain the differences in results (9–70 number of sprint), and it was repeated in male studies as well [16]. In relation to the ACC and DEC actions, these variables can be strongly influenced by the device used and its sensitivity, as well as the duration of the action to be considered as ACC or DEC (i.e. 2–3 seconds) [119]. Studies [32–35, 41, 43, 54, 56, 63, 65] revealed that players performed a range of 8–423 and 15–430 in ACC-DEC actions per match respectively, while male soccer players performed about 64 ACC and 58 DEC actions per match (2–3 m × s^−1^) [120]. Knowledge of the demands of elite women’s soccer matches can be very useful for coaches, physical trainers, and physiotherapists to plan tailor-made training and return-to-play sessions.

In rugby league and union very similar TD were reported, with a mean of ~5533 m [72, 75, 87] and ~5458 m [71, 73, 74, 76] respectively. Considering TD performed per minute, the rugby league players performed ~77 m × min^−1^ and the rugby union players about ~65 m × min^−1^. In rugby sevens TD was ~1549 m [76–79, 81, 82, 84–86, 90, 91], ~72% lower than rugby league and union; however, when reported relative to time it was slightly higher at 94 m × min^−1^. Considering distances, female rugby league and union players covered 934 m and 114 m in MSR and HSR, respectively, whilst sevens elite female players performed 355 m in MSR, 165 m in HSR, and 108 m in sprinting. A recent meta-analysis [121] found that male sevens players covered 1100–2486 m of TD, 77–121 m × min^−1^, ~449 m in MSR and ~190 m in HSR – greater distance than women players, especially at high speeds. The same was observed in rugby league and union male players, who performed greater distances [122, 123]. Female rugby players completed a mean of 7 and 5 sprints per match in rugby league/union and rugby sevens respectively. The variability of results may be explained by positional differences of rugby demands (i.e., backs, forwards) and the differences in the sports’ rules and discipline. Therefore, reference values from different rugby disciplines are important, especially when players interchange within rugby sports, or return to play following a long-term injury or illness.

In field hockey, TD covered was similar in studies, ~5403 m [94–99, 101], of which ~823 m were in MSR, ~466 m in HSR and ~371 m in sprinting. Slightly lower results were found by James et al. [124] in male players (TD = ~4861 m; > 14.5 km × h^−1^ = ~1193 m; > 19 km × h^−1^ = ~402 m). Elite female field hockey players performed a mean of ~39 sprints, ~26 ACC and ~32 DEC actions; however, male field hockey players [124] reported that they performed ~21 sprints, ~50 ACC and ~60 DEC actions per match. Coaches and physical trainers may know the demands that competition requires, and in consequence these values can help to better understand the efforts that hockey players make during the competition. This would make it possible to compare the physical level with elite hockey reference values and draw the lines of work for both conditioning and recovery; however, more research is needed.

In female basketball, the TD covered was 7039 m, using TMA [82], and similar results were obtained for male players in a systematic review [125] (TD = ~7558 m) when the same system was used. Reina et al. [109] used LPS and found that women players covered 3531 m. The studies indicated that the proportion of movement performed by female basketball players was: MSR ~16%, HSR ~7%, and sprinting ~7%; while male players covered ~40% in MSR, ~25% in HSR, and ~0.4% in sprinting [125]. Also, elite female basketball players did ~35 sprint actions per match [105, 108]. Although few studies are available, these values can help to better understand the demands of elite women’s basketball, and further investigation is warranted.

Regarding handball demands, studies that used TMA analysis reported that TD ranged between 2882 and 4002 m [114, 115]. Similar results were found in male players (i.e., ~3.5 km) [126, 127]. Elite female handball players covered ~423 m in MSR and ~141 m in HSR; similarly, during professional men’s matches, players covered 356–670 m in MSR and 133–153 m in HSR. Moreover, the range of Pload was 8.8–10.6 au × min^−1^ [110–113] and women players performed 8.7–2.3 ACC and DEC per minute respectively.

Considering futsal, only one study [116] recorded female futsal match demands, using LPS. Players ran an average of ~5 m × min in HSR, with a threshold close to 20 km × h^−1^. In addition, approximately ~0.4 ACC per minute of play (> 4–5 m × s^−2^) were performed, the maximum ACC was 6 m × s^−2^ and 240 m × min^−1^ were covered in ACC, which corresponds to a total of ~28 ACC × min^−1^. The maximum DEC was ~-6 m × s^−2^ and ~28 DEC × min^−1^ was performed. However, male futsal players presented higher match demands when compared to female futsal players [29]. Given that, methods and strategies in female’s team sports should not be supported by evidence derived from male athletes.

There is limited evidence available regarding external load monitoring in indoor sports. This could be attributed to the fact that many indoor sports are practised in confined spaces, which makes it challenging to use tracking and monitoring devices compared to outdoor sports (due to e.g. high cost, complex installation, variables) [128, 129]. Each tracking technology has unique approaches to monitoring athletes, resulting in distinct advantages and disadvantages when tracking external load; therefore, it is essential to consider how the technology and its manufacturer process data within the context of the sport [11].

It should be noted that there are a number of contextual factors (i.e., team characteristics, style of play, opponent characteristics, moods, starter/non-starter, competition situations and venue) that may have influenced the variability of the data [130, 131]. The context can significantly affect the performance of the players and, therefore, the results obtained through the tracking system. It is important for staff to consider these variables when analysing the demands of competition and the variation that can occur from match to match. Therefore, it is recommended to avoid drawing absolute conclusions from a single measurement and instead analyse multiple data points to gain an overall understanding of the demands of competition.

On the other hand, this study established specific speed ranges for MSR, HSR, and sprinting to simplify the summary and comparison of results regarding the distance covered. However, the selection of speed thresholds lacks consensus, particularly regarding external load monitoring with wearable devices for female athletes. While most studies have focused on male athletes, some have suggested that speed thresholds set for men may not be applicable for women due to underestimation of efforts and inaccuracy of results [86, 132, 133]. Therefore, the authors recommend using relative thresholds in monitoring with wearable devices for better interpretation of results. Considering individual athlete performance and the use of absolute thresholds allows for a broader comparison and establishment of general goals [86, 134–136]. Consequently, further evidence is needed to determine whether female athletes require a different external load control approach than male athletes and whether it differs between sports.

Another point to consider is the definition of “elite” status in sports, which is a complex issue depending on several factors [137]. Generally, elite athletes are those who have achieved a high level of performance in their sport and compete at a professional level or in international competitions; criteria such as world ranking in a given sport discipline, history of achievement in major competitions, Olympic medal winning, or participation in national teams could be used [138, 139]. Nonetheless, defining elite status in sport can be challenging because it can vary depending on the sport and country in question [137]. Additionally, the level of performance required to be considered an elite athlete may change over time as sports evolve and athletes become stronger and faster [140, 141].

This study is limited by the lack of consistency of the devices (i.e., GPS, TMA, LPS), thresholds of different actions (i.e. zones of intensity, sprint, ACC, DEC), and sampling frequencies (1–15 Hz) that have been used. Lower sampling frequencies (e.g. 1 Hz, 5 Hz) have been shown to be less reliable than 10 Hz [119, 142], whereas with 10 Hz, the occurrence of high-intensity ACC and DEC actions can be obtained reliably, although distance and time-related variables are less reliable [119, 143]. The data filtering technique used by different software and upgrades can also influence the quality, reliability, and usefulness of the data [143, 144]. In addition, the minimum time that an ACC or DEC action must stabilize above the threshold to be determined as effort could generate inaccuracies in the frequencies of ACC and DEC of greater intensity [145]. Depending on the variables analysed, in elite female athletes, analysing between 3 and 9 matches, less than 10% error was found for profiling [146]. Finally, the present study did not consider positional differences or other variables (e.g., impacts, ACC and DEC zones, and peak velocity, among others) that might be of interest. Therefore, practitioners and researchers should carefully consider the methodology used and the criteria used to delineate the variables of interest.

## CONCLUSIONS

In conclusion, this systematic review provides information regarding the match demands of elite female team sports. Soccer is the most investigated sport; female players perform ~9500 m TD; also they do ~580 m in HSR with a great number of ACC, DEC, and sprints. Rugby league and union players cover a greater distance (~5450 m) when compared to rugby sevens (~1550 m); however, rugby sevens is more demanding in terms of high-intensity actions. Women’s field hockey players perform ~5400 m TD; also, it is a high-intensity sport, with high-speed and sprint actions. Women’s indoor sports are less studied, which could be due to the difficulty and high cost of measuring the external load indoors. Female basketball players cover ~5300 m TD, of which 7% are in MSR. In handball, elite women’s players perform ~3500 m TD; also, they cover ~423 m in MSR and ~141 m in HSR. Finally, female elite futsal players perform ~5 m × min^−1^ in HSR and they do a great number of high-intensity activities (i.e., HSR, ACC, and DEC actions). We consider that the results obtained from the existing research on the competitive demands of female athletes in team sports should be considered as a starting point, while keeping in mind the limitations discussed earlier. Additionally, it is important to customize the methods for external load monitoring based on the particular context and objectives of each sport. Lastly, we strongly recommend that researchers and professionals continue to explore and expand the knowledge on external load monitoring in female athletes.
